# Supplementary material for: Estimation of Linkage Disequilibrium, Effective Population Size, and Genetic Parameters of Phenotypic Traits in Dabieshan Cattle
Source: Genes (Basel). 2022 Dec 29;14(1):107. doi: 10.3390/genes14010107 (PMC9859230; doi:10.3390/genes14010107)
Supplement: Supplementary file 1 [file genes-14-00107-s001.zip › genes-2037457-supplementary/Supplementary Figure S1 Number of SNPs in each autosome for GGP 100K before and after SNP quality control (QC).pdf]

Supplementary Figure S1 Number of SNPs in each autosome for GGP 100K before and after SNP quality control (QC)

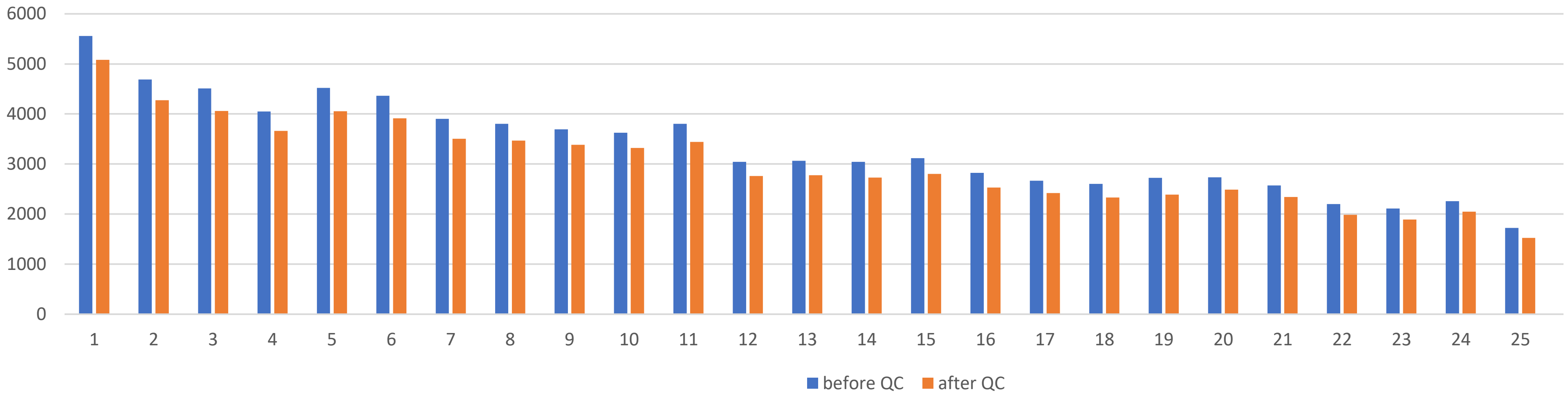

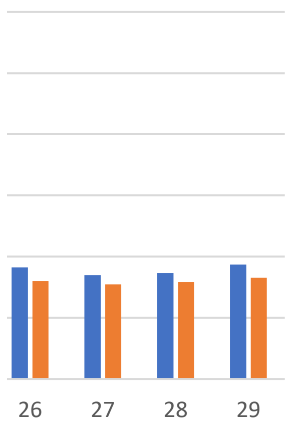
26
27
28
29
