# Supplementary material for: Estimation of Linkage Disequilibrium, Effective Population Size, and Genetic Parameters of Phenotypic Traits in Dabieshan Cattle
Source: Genes (Basel). 2022 Dec 29;14(1):107. doi: 10.3390/genes14010107 (PMC9859230; doi:10.3390/genes14010107)
Supplement: Supplementary file 1 [file genes-14-00107-s001.zip › genes-2037457-supplementary/Supplementary Table S1 Number of SNPs in each autosome for GGP 100K before and after SNP quality control in DBSC.pdf]

Supplementary Table S1 Number of SNPs in each autosome for GGP 100K before and after SNP quality control in DBSC

chr	Bos taurus ARS-UCD1.3 chr length (Mb)	before quality control			after quality control		
		SNP Num	SNP percentage(%)	average interval (kb)	SNP Num	SNP percentage(%)	average interval (kb)
1	158.53	5556	6.15	28.53	5082	5.62	31.19
2	136.23	4688	5.19	29.06	4273	4.73	31.88
3	121.01	4508	4.99	26.84	4061	4.49	29.80
4	120.00	4049	4.48	29.64	3660	4.05	32.79
5	120.09	4523	5.01	26.55	4055	4.49	29.62
6	117.81	4364	4.83	27.00	3913	4.33	30.11
7	110.68	3903	4.32	28.36	3504	3.88	31.59
8	113.32	3805	4.21	29.78	3470	3.84	32.66
9	105.45	3695	4.09	28.54	3383	3.74	31.17
10	103.31	3626	4.01	28.49	3321	3.68	31.11
11	106.98	3801	4.21	28.15	3442	3.81	31.08
12	87.22	3044	3.37	28.65	2761	3.06	31.59
13	83.47	3064	3.39	27.24	2776	3.07	30.07
14	82.40	3045	3.37	27.06	2727	3.02	30.22
15	85.01	3119	3.45	27.26	2804	3.10	30.32
16	81.01	2826	3.13	28.67	2532	2.80	31.99
17	73.17	2668	2.95	27.43	2420	2.68	30.24
18	65.82	2605	2.88	25.27	2332	2.58	28.22
19	63.45	2726	3.02	23.28	2389	2.64	26.56
20	71.97	2737	3.03	26.30	2490	2.76	28.90
21	69.86	2573	2.85	27.15	2342	2.59	29.83
22	60.77	2201	2.44	27.61	1986	2.20	30.60
23	52.50	2110	2.34	24.88	1891	2.09	27.76
24	62.32	2259	2.50	27.59	2050	2.27	30.40
25	42.35	1726	1.91	24.54	1523	1.69	27.81
26	51.99	1823	2.02	28.52	1602	1.77	32.45
27	45.61	1699	1.88	26.85	1546	1.71	29.50
28	45.94	1735	1.92	26.48	1586	1.76	28.97
29	51.10	1871	2.07	27.31	1658	1.84	30.82
sum	2489.37	90349	100.00	27.55	81579	90.29	30.51

Quality control criteria and codes were: plink – cow – file genotypefile_ name – geno 0.1 – maf 0.05 – hwe 10-6 – mind 0.1 – recode12 – out filename.

BEAGLE running command was: java – Xmx1000m – jar beagle.jar unphased = file.bgl missing = 0 out = example niterations =100.
