# Supplementary material for: Estimation of Linkage Disequilibrium, Effective Population Size, and Genetic Parameters of Phenotypic Traits in Dabieshan Cattle
Source: Genes (Basel). 2022 Dec 29;14(1):107. doi: 10.3390/genes14010107 (PMC9859230; doi:10.3390/genes14010107)
Supplement: Supplementary file 1 [file genes-14-00107-s001.zip › genes-2037457-supplementary/Supplementary Table S2 The details descriptive results of markers and LD (r2) between every 2.5kb adjacent markers across autosomes.pdf]

Supplementary Table S2 The details descriptive results of markers and LD (r2) between every 2.5kb adjacent markers across autosomes

dist	r2
2.5	0.657642
5	0.579005
7.5	0.563553
10	0.53371
12.5	0.52541
15	0.519215
17.5	0.510808
20	0.499905
22.5	0.488238
25	0.483249
27.5	0.468719
30	0.460787
32.5	0.454692
35	0.451239
37.5	0.445408
40	0.442685
42.5	0.440492
45	0.431512
47.5	0.428473
50	0.420373
52.5	0.418243
55	0.421644
57.5	0.415158
60	0.417063
62.5	0.397845
65	0.40527
67.5	0.408541
70	0.405946
72.5	0.400953
75	0.389029
77.5	0.384452
80	0.388864

82.5	0.38331
85	0.381528
87.5	0.387508
90	0.38565
92.5	0.387398
95	0.378838
97.5	0.38359
100	0.365458
102.5	0.364928
105	0.365586
107.5	0.365151
110	0.361384
112.5	0.363935
115	0.353847
117.5	0.363926
120	0.359054
122.5	0.359245
125	0.354446
127.5	0.350471
130	0.340488
132.5	0.359477
135	0.350118
137.5	0.353354
140	0.352236
142.5	0.352291
145	0.350316
147.5	0.351567
150	0.33968
152.5	0.355686
155	0.336376
157.5	0.34326
160	0.347956
162.5	0.348459
165	0.339766
167.5	0.342539

170	0.335359
172.5	0.340846
175	0.336272
177.5	0.327033
180	0.333189
182.5	0.331752
185	0.325626
187.5	0.331503
190	0.332648
192.5	0.329917
195	0.318028
197.5	0.33944
200	0.332342
202.5	0.324536
205	0.321134
207.5	0.32612
210	0.316612
212.5	0.325654
215	0.31862
217.5	0.327149
220	0.315589
222.5	0.325407
225	0.314247
227.5	0.320299
230	0.311256
232.5	0.315864
235	0.324123
237.5	0.310767
240	0.321353
242.5	0.314916
245	0.309528
247.5	0.323379
250	0.319603
252.5	0.310477
255	0.315293

257.5	0.305025
260	0.31122
262.5	0.324691
265	0.315405
267.5	0.302776
270	0.313682
272.5	0.317892
275	0.308151
277.5	0.313174
280	0.313549
282.5	0.311033
285	0.308974
287.5	0.311743
290	0.315264
292.5	0.309336
295	0.313843
297.5	0.300615
300	0.298056
302.5	0.303475
305	0.308389
307.5	0.306759
310	0.310441
312.5	0.302245
315	0.302424
317.5	0.293226
320	0.302256
322.5	0.31694
325	0.295221
327.5	0.304418
330	0.295308
332.5	0.304405
335	0.290038
337.5	0.297765
340	0.311274
342.5	0.314736

345	0.2961
347.5	0.301789
350	0.283673
352.5	0.295068
355	0.293093
357.5	0.300536
360	0.299436
362.5	0.310731
365	0.297986
367.5	0.295688
370	0.301713
372.5	0.305494
375	0.300723
377.5	0.30775
380	0.29931
382.5	0.303292
385	0.301906
387.5	0.297366
390	0.293597
392.5	0.296124
395	0.289114
397.5	0.283687
400	0.30107
402.5	0.290923
405	0.284729
407.5	0.284716
410	0.293958
412.5	0.291453
415	0.307777
417.5	0.290803
420	0.289871
422.5	0.290229
425	0.298311
427.5	0.2854
430	0.294869

432.5	0.290862
435	0.293546
437.5	0.286183
440	0.28604
442.5	0.278602
445	0.27847
447.5	0.282925
450	0.283184
452.5	0.278791
455	0.289627
457.5	0.284425
460	0.276978
462.5	0.308208
465	0.291262
467.5	0.273105
470	0.282599
472.5	0.275851
475	0.287185
477.5	0.27422
480	0.29443
482.5	0.29295
485	0.288626
487.5	0.284641
490	0.287063
492.5	0.280148
495	0.282049
497.5	0.27523
500	0.287629
502.5	0.281321
505	0.272532
507.5	0.274088
510	0.289481
512.5	0.277234
515	0.283754
517.5	0.290475

520	0.283431
522.5	0.27279
525	0.27962
527.5	0.304306
530	0.293644
532.5	0.289258
535	0.284294
537.5	0.289491
540	0.293707
542.5	0.270006
545	0.262694
547.5	0.286066
550	0.276754
552.5	0.282229
555	0.28931
557.5	0.280756
560	0.268499
562.5	0.282218
565	0.311068
567.5	0.285374
570	0.26541
572.5	0.267826
575	0.256533
577.5	0.292581
580	0.266869
582.5	0.295698
585	0.267337
587.5	0.264151
590	0.298713
592.5	0.274276
595	0.275044
597.5	0.266803
600	0.266995
602.5	0.280221
605	0.278871

607.5	0.301244
610	0.292358
612.5	0.293706
615	0.266648
617.5	0.289387
620	0.259454
622.5	0.264301
625	0.291414
627.5	0.281692
630	0.288761
632.5	0.257231
635	0.286546
637.5	0.279015
640	0.274412
642.5	0.262662
645	0.293597
647.5	0.286128
650	0.287149
652.5	0.271466
655	0.270706
657.5	0.265867
660	0.272977
662.5	0.286693
665	0.272516
667.5	0.28704
670	0.271773
672.5	0.278951
675	0.301209
677.5	0.265237
680	0.284502
682.5	0.280432
685	0.281834
687.5	0.276078
690	0.257891
692.5	0.277397

695	0.277001
697.5	0.256273
700	0.272769
702.5	0.29372
705	0.300443
707.5	0.279931
710	0.284152
712.5	0.269456
715	0.263792
717.5	0.25565
720	0.281419
722.5	0.276227
725	0.259278
727.5	0.279427
730	0.256297
732.5	0.273482
735	0.268061
737.5	0.263693
740	0.266463
742.5	0.261677
745	0.268446
747.5	0.257308
750	0.253727
752.5	0.275321
755	0.266999
757.5	0.299942
760	0.292279
762.5	0.265506
765	0.277007
767.5	0.25377
770	0.279715
772.5	0.25023
775	0.259598
777.5	0.254024
780	0.278358

782.5	0.265667
785	0.26915
787.5	0.245875
790	0.280387
792.5	0.269755
795	0.259931
797.5	0.249731
800	0.263372
802.5	0.253795
805	0.276896
807.5	0.266745
810	0.269006
812.5	0.249429
815	0.255472
817.5	0.258109
820	0.263045
822.5	0.292845
825	0.246075
827.5	0.276756
830	0.27877
832.5	0.271124
835	0.269395
837.5	0.241126
840	0.303027
842.5	0.243023
845	0.261669
847.5	0.283701
850	0.293933
852.5	0.258724
855	0.275285
857.5	0.274785
860	0.289861
862.5	0.255581
865	0.272481
867.5	0.250422

870	0.282401
872.5	0.275033
875	0.270032
877.5	0.273753
880	0.250427
882.5	0.237652
885	0.294366
887.5	0.270911
890	0.276746
892.5	0.242356
895	0.271267
897.5	0.265036
900	0.255325
902.5	0.284894
905	0.278142
907.5	0.252795
910	0.268564
912.5	0.264336
915	0.244608
917.5	0.275911
920	0.275788
922.5	0.268071
925	0.262677
927.5	0.260932
930	0.275686
932.5	0.24619
935	0.287561
937.5	0.250467
940	0.27535
942.5	0.265291
945	0.243967
947.5	0.29154
950	0.248721
952.5	0.24778
955	0.26967

957.5	0.262432
960	0.255243
962.5	0.249661
965	0.234964
967.5	0.328438
970	0.273206
972.5	0.292838
975	0.241211
977.5	0.269782
980	0.282733
982.5	0.280211
985	0.242142
987.5	0.272346
990	0.242571
992.5	0.292049
995	0.263538
997.5	0.274607
1000	0.303301
