# Supplementary material for: Estimation of Linkage Disequilibrium, Effective Population Size, and Genetic Parameters of Phenotypic Traits in Dabieshan Cattle
Source: Genes (Basel). 2022 Dec 29;14(1):107. doi: 10.3390/genes14010107 (PMC9859230; doi:10.3390/genes14010107)
Supplement: Supplementary file 1 [file genes-14-00107-s001.zip › genes-2037457-supplementary/Supplementary Table S3 Summary of SNP pairs, average, standard deviation (SD), median linkage disequilibrium (r2) for DBSC.pdf]

Supplementary Table S3 Summary of SNP pairs, average, standard deviation (SD), median linkage disequilibrium (r2) for DBSC

Distance	SNP pairs	Average r-squared	SD	Median
0-2.5	3075	0.7035704	0.2835458	0.775999
2.5-5	2687	0.5790053	0.2636156	0.535473
5-7.5	2442	0.5635531	0.2614677	0.524966
7.5-10	2319	0.5337099	0.2524151	0.477849
10-20	8719	0.5139892	0.2409004	0.455882
20-30	9024	0.4758339	0.2232411	0.4170135
30-40	7468	0.4486344	0.2081659	0.3922125
40-50	6605	0.4303838	0.1983906	0.371716
50-60	5882	0.4180244	0.1909446	0.359009
60-70	5369	0.4043249	0.1824611	0.348207
70-80	4821	0.391079	0.1705185	0.340922
80-90	4444	0.3844768	0.1697847	0.329926
90-100	4098	0.3790431	0.1650969	0.3274005
100-200	27032	0.3483698	0.1460969	0.301506
200-300	13927	0.3159687	0.1177494	0.277404
300-400	7927	0.3007354	0.1051323	0.267144
400-500	4770	0.2872581	0.0941063	0.256381
