# Supplementary material for: Estimation of Linkage Disequilibrium, Effective Population Size, and Genetic Parameters of Phenotypic Traits in Dabieshan Cattle
Source: Genes (Basel). 2022 Dec 29;14(1):107. doi: 10.3390/genes14010107 (PMC9859230; doi:10.3390/genes14010107)
Supplement: Supplementary file 1 [file genes-14-00107-s001.zip › genes-2037457-supplementary/Supplementary Table S4 The descriptive statistical results of each trait for DBSC.pdf]

Supplementary Table S4 The descriptive statistical results of each trait for DBSC

Phenotype Trait	Num	Average	SD	Min	Max	Medium	Coefficient of Variation (%)
wither height (WH)	235	110.23	7.01	102	130	111	6.36%
height at hip cross (HHC)	235	111.11	6.72	99	129	112	6.04%
diagonal body length (DBL)	235	122.86	10.43	117	156	124	8.49%
chest girth (CG)	235	154.56	19.23	140	224	155	12.44%
abdomen circumference (AC)	235	182.33	22.67	149	275	180	12.43%
waist angle width (WAW)	235	35.63	4.71	26	45	36	13.21%
ischial end width (IEW)	235	16.40	3.25	8	46	16	19.83%
shin circumference (SC)	219	16.41	1.82	14	21	16	11.07%
body weight (BW)	223	270.85	50.50	237	425	263	18.65%

unit, cm or Kg
