# Supplementary material for: Estimation of Linkage Disequilibrium, Effective Population Size, and Genetic Parameters of Phenotypic Traits in Dabieshan Cattle
Source: Genes (Basel). 2022 Dec 29;14(1):107. doi: 10.3390/genes14010107 (PMC9859230; doi:10.3390/genes14010107)
Supplement: Supplementary file 1 [file genes-14-00107-s001.zip › genes-2037457-supplementary/Supplementary Table S5 Significance of the fixed effects included in the linear model.pdf]

Supplementary Table S5 Significance of the fixed effects included in the linear model

Fixed effects including farm, birth year, and gender using the following equation:

$$y_{ijklmn} = u + Farm_i + Year_j + Sex_k + e_{ijklmn}$$

Where y_{ijklmn} is the phenotypic value, u is the population mean, $Farm_i$, $Year_j$, and Sex_k are the effect of farm, birth year, and gender, respectively. e_{ijklmn} is a random residual.

Supplementary Table S5 Significance of the fixed effects included in the linear model

Fixed effects	farm	birth year	gender
Wither Height (WH)	**	**	**
Height at Hip Cross (HHC)	**	NS	**
Diagonal Body Length (DBL)	**	**	**
Chest Girth (CG)	**	NS	**
Abdomen Circumference (AC)	NS	**	**
Waist Angle Width (WAW)	**	NS	**
Ischial End Width (IEW)	**	**	**
Shin Circumference (SC)	NS	NS	**
Body Weight (BW)	**	NS	**

Note: NS =nonsignificant, **P<0.05, *P<0.1.
